# One must reconstitute the functions of interest from purified proteins

**DOI:** 10.3389/fphys.2024.1390186

**Published:** 2024-05-17

**Authors:** James A. Spudich

**Affiliations:** Department of Biochemistry, Stanford University School of Medicine, Stanford, CA, United States

**Keywords:** myosin, actin, *Dictyostelium*, motility assay, single molecule assay, interdisciplinary research

## Abstract

I am often asked by students and younger colleagues and now by the editors of this issue to tell the history of the development of the *in vitro* motility assay and the dual-beam single-molecule laser trap assay for myosin-driven actin filament movement, used widely as key assays for understanding how both muscle and nonmuscle myosin molecular motors work. As for all discoveries, the history of the development of the myosin assays involves many people who are not authors of the final publications, but without whom the assays would not have been developed as they are. Also, early experiences shape how one develops ideas and experiments, and influence future discoveries in major ways. I am pleased here to trace my own path and acknowledge the many individuals involved and my early science experiences that led to the work I and my students, postdoctoral fellows, and sabbatical visitors did to develop these assays. Mentors are too often overlooked in historical descriptions of discoveries, and my story starts with those who mentored me.

## Mentors are important players in all of our lives

**FIGURE 1:**
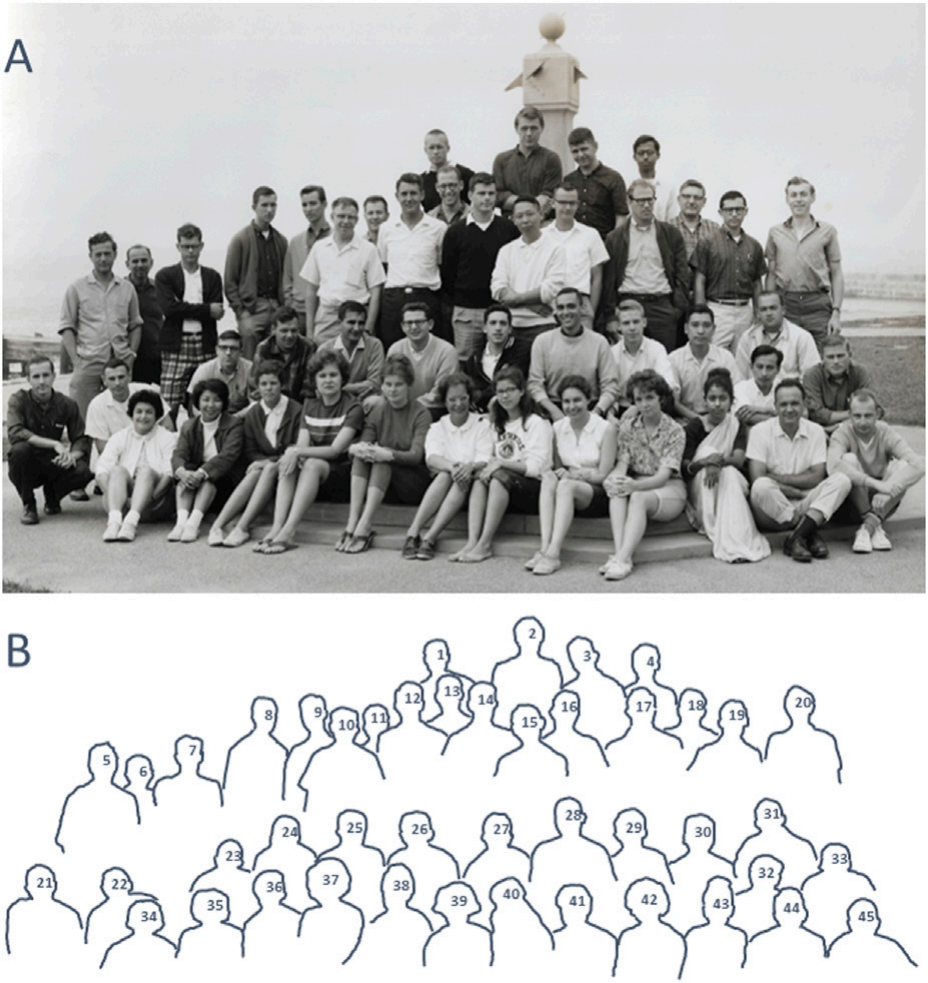
Participants in the Woods Hole 1963 summer Physiology Course. **(A)** Photograph of participants. Not all participants are in the photo. **(B)** Pen outline of the individuals in the photo, numbered to coincide with the numbers in parentheses below. A complete list of course participants is: *Course Director and Instructor*–J. Woodland Hastings (12). Instructors–Alex Keynan (6), Harlyn Halvorson (10), Shinya Inoue (15), Fred Karush (18), Ken van Holde (24), Ed Adelberg, Philip Hartman. Assistant Instructors–with Hastings, Jim Spudich (9), Jorge Churchich (25), Margarita Churchich; with van Holde, William Deal (11), Lawrence Cohen, S. Y. Sun; with Halvorson, Roger Bretthauer (33); with Inoue, Hidemi Sato (30), Jonathan Hardy (2); with Hartman and Adelberg, M. Levin (22), Riyo Kunisawa (35), Frank Vasington; with Karush, Sayaka Utsumi (32). *Course Assistants*–Carolyn Veeder (40), John Spudich (8), Randy Sweeney. *Consultants, Guest lecturers*–Merkel Jacobs, Arthur Parpart, Albert Szent-Gyorgyi, William McElroy, Martin Pollock (5?), Herman Kalckar (5?). *Students*–Robert Barlow, Jr (1), Kunal Saha (4), Jon Jacklett (7), Jay Mittenthal (13), Joel Shaper (13), Raymond Stephens (16), William Hahn (17), Ben Leichtling (19), Deric Bownds (21), Allen Phillips (23), Robert Trivus (26), Steve Harrison (27), Joseph Fratantoni (28), Robert Grey (29), Marshall Elzinga (31), Laura Ponticorvo (34), Dorothy Nauman (36), Shirley Hilden (37), Mary-Jane Tunis (38), Linda Garrick (39), Yvonne Connelly (41), Pam Stoddard (42), Annamma Cownan (43), Felix Madrid (44), Richard Humphrey (45), Julian Haynes, Eugene Jacobson, Ronald Sederoff, Willie Curry, Richard DeSa, Richard Muesing. Source: Reproduced from Marine Biological Laboratory Archives, licensed under CCBY 4.0 International.

My own story could start as a boy fascinated with chemistry, but I will fast forward to my experiences in the Physiology Course in Woods Hole, during the summers of 1962 and 1963, where I learned the importance of interdisciplinary approaches for elucidating questions about a biological system. The interdisciplinary approach, widely used in laboratories now, was not common in an individual laboratory or even in a department then. The Physiology Course in 1962 and 1963 was headed up by my first mentor, John Woodland Hastings, known as “Woody,” with whom I worked on bioluminescence as an undergraduate chemistry major at the University of Illinois, Urbana-Champaign. Woody was ahead of his time assembling an interdisciplinary group of stellar course instructors covering biochemistry, physics, genetics, developmental biology, and cell biology. Instructors came from far and wide. Ken van Holde was a physical biochemist at the University of Illinois, who together with Robert “Buzz” Baldwin, developed a method for rapid approach to sedimentation equilibrium and its use to analyze the size and shape of protein molecules. Ken van Holde also designed improvements in light scattering and circular dichroism. In the course, he taught about basic chemical and physical properties of proteins in relation to their bioIogical specificity and function. The laboratory studies included investigations of the physical properties of protein molecules which are sensitive to their molecular configuration, weight, and shape. Phil Hartman was a Johns Hopkins Biologist and pioneer in microbial genetics and mutagenesis. Ed Adelberg, from Yale, was a founder of microbial genetics. One of his long-term investigations involved the genetic regulation of amino acid biosynthesis in *E. coli*. In the course, Phil Hartman and Ed Adelberg taught microbial genetics and physiology. The lab involved isolation of bacterial mutants, analysis of growth factor requirements, and genetic analysis through transduction tests as well as by conjugation. Alex Keynan was an Israeli microbiologist who studied germination of *Bacillus subtilis* spores and quorum sensing in bacterial populations. Harlyn Halvorson, who later served as Director of the Marine Biological Laboratory, was a microbiologist who also studied bacterial sporulation. Two of my favorite instructors were the biochemist Andrew Szent-Györgyi, who introduced me to muscle research, and the cell biologist Shinya Inoue, who was developing his latest innovations in light microscopy for studying spindle dynamics during mitosis. Their influence on the specifics of my future scientific contributions was pivotal. Imagine as a young scientist in training, just completing his B.S. degree, being exposed to such an intense course with a diverse array of approaches to biological problems. I had published three manuscripts from my undergraduate work with Woody, combining my organic chemistry expertise with biochemistry and photometry. Those were exciting times for me, and the Woods Hole Physiology Course then and now changes the scientific lives of many students who experience it (Figure 1).

Leaving Woods Hole in 1963, I began my graduate training in the Department of Biochemistry at Stanford, established by Arthur Kornberg. I was in a class of three new graduate students and was one of the earliest students in the Department, which was founded just 4 years earlier. The ratio of students to faculty at that time was close to 1:1. One of the many remarkable aspects of the department was that, although Arthur was my thesis advisor, all the faculty members, Paul Berg, Bob Lehman, Dale Kaiser, Buzz Baldwin, Dave Hogness, and the new assistant professors Lubert Stryer and George Stark, were all my mentors. The Department was heavily focused on hard-core biochemistry–you ground up cells, made an extract, which was then fractionated by conventional biochemical approaches, following the activity of the enzyme you were trying to purify*. “You will never understand how a system works if you cannot reconstitute the functions of interest from purified proteins!”* was drilled into us graduate students–this was the Department of Biochemistry’s creed. The Department was so focused on protein purification from cell extracts that there was not even a microscope available when I started. To visualize *B. subtilis* cells, which I was happily grinding up in pursuit of the origin of proteins in the developmental transition from a vegetative cell to a spore, I had to borrow a microscope from the Department of Genetics. Shinya Inoue would have been amused.

Those years of hard-core biochemistry focus and training were invaluable throughout my career and taught me many fundamental facts and principles that I would not have learned otherwise. Arthur was interested in starting a program on understanding the developmental transition of a *B. subtilis* vegetative cell into a spore. I knew a lot about that subject from my Woods Hole experience and was delighted to help launch that new program in his lab. Early on in my thesis research, I purified *B. subtilis* adenylate kinase from vegetative cells and spores to compare their properties. After purifying over multiple columns, I had activity in a very low protein concentration fraction, but in the next step I lost all activity! This could have been due to the loss of a critical co-factor, as Tom Pollard and Ed Korn describe in an accompanying paper in this issue (Pollard and Korn, 2023). But in my case, it was another key lesson that all biochemists learn–placing a protein in a glass or plastic tube immediately results in the protein adhering to the surface of the vessel as a monolayer. In a very dilute solution of protein at a near final purification step, one can lose all your enzyme stuck onto the tube surface, which is what happened to my laboriously purified adenylate kinase. A lesson hard learned–one must rinse glass- and plastic-ware with a bovine serum albumin (BSA) solution to saturate the surface with protein to avoid losing your precious purified enzyme. A nascent glass or plastic surface tightly binds a monolayer of the first protein presented to it. I would use this hard-earned knowledge, 5 years later in my laboratory, to great eventual benefit, as a beginning Assistant Professor in the new Department of Biochemistry and Biophysics at UCSF chaired by Bill Rutter.

When in 1968, it was time to start post-doctoral work, my intuition derived from my Woods Hole experience led me in a very different direction from the common practice of graduate students at the time. The usual practice was a 1-yr postdoctoral experience working on a very similar area of research as a graduate student, but in Europe to get the “European experience,” and then assume an assistant professorship back in the US continuing research on the same problem using the same familiar biochemical approaches. I was, however, determined to obtain first-hand exposure to both genetics and structural biology, and therefore planned two consecutive postdocs, totaling 3 years of postdoctoral research, nearly unheard of in those days. Arthur said I would be an old man before starting creative research on my own, but my instincts to get firsthand experiences in genetics and structural biology proved to be a decision that would serve me well in my research career.

I arranged to spend the year of 1968 with Charley Yanofsky in the Department of Biology at Stanford using genetic approaches, working to understand recombination near the tryptophan operon in *E. coli*. That year, working with another giant of a scientist, using purely genetic approaches, had an enormous impact on my research going forward. We published an interesting paper regarding DNA recombination in the Journal of Molecular Biology from those studies.

My next step was to learn techniques of structural biology. In those days there was only one place to consider going to learn structural biology, the Laboratory of Molecular Biology (LMB) at the Medical Research Council (MRC) in Cambridge, England. This powerhouse of Nobel laureates was an inspiring place, several miles outside of Cambridge center. Being isolated, a restaurant with long rectangular tables was established on the sixth floor of the building where lunch was served as well as afternoon tea. This is where an enormous amount of scientific exchange occurred between various laboratories, and new ideas emerged daily. That sixth floor restaurant was essential for the dynamic cross-fertilization that was so impressive there, and I was to incorporate this into an initiative that I became involved in 30 years later at Stanford called Bio-X (https://biox.stanford.edu/; for early history, see Supplementary Information).

At the LMB, Aaron Klug was at an early stage of carrying out reconstructions from electron micrographs to obtain the 3D structure of virus particles. Working with John Finch and David DeRosier, Aaron showed that in cases like tobacco mosaic virus, being a long cylindrical, helical structure, one can use helical diffraction theory to solve its structure, where single views of the virus contain all the information needed to obtain the 3D structure (De Rosier and Klug, 1968). Think of a barber pole–from one view from one direction you can understand the 3D structure of the barber pole pattern. Rather than apply directly to Aaron’s lab for a postdoc position, my interests in muscle contraction, peaked by interactions with Andrew Szent-Györgyi in Woods Hole, led me to join the laboratory of Hugh Huxley, the leader in the field of muscle structure and function. Peter Moore, Hugh Huxley and David DeRosier were just publishing a helical reconstruction of actin with myosin heads bound when I arrived in Cambridge in 1969 (Moore et al., 1970). It was also the year that Hugh was submitting for publication his pivotal *Science* paper on the swinging crossbridge model of muscle contraction (Huxley, 1969), a draft of which he gave me to read upon my arrival. My previous training was so focused on biochemistry and genetics that I remember being captivated by the power of the structural approaches Hugh used, both electron microscopy of fixed muscle and isolated proteins and low angle X-ray scattering of live muscle tissue, to reveal important concepts of how muscle works. From the perspective of my background, however, I was surprised that there was no biochemical *in vitro* reconstitution of the primary functions of interest, which in this case are *movement and force production.* Furthermore, genetic approaches were largely missing. Both became focuses of my own laboratory after I left the LMB for my first faculty position at UCSF.

As a postdoctoral fellow at the LMB, I was keen to contribute something new to Hugh’s lab and to the field. At the time, it was clear that the tropomyosin-troponin complex was important for calcium regulation of muscle contraction, but the biochemical mechanism and structural aspects of the system were not worked out. My postdoctoral work at LMB necessarily began with purification of the proteins involved, actin and tropomyosin-troponin, so I could study their interaction. Actin preparations at that time were heavily contaminated with tropomyosin-troponin, and because of my description in my first paper from this work of how to obtain highly purified actin, it became one of my most frequently cited papers (Spudich and Watt, 1971). The actin purification method overshadowed the important biochemical titrations described in that paper that proved that tropomyosin-troponin regulates the actin-myosin interaction by complexing with actin, and in a 1:7 M ratio of tropomyosin-troponin:actin. A key associate of Hugh’s at the LMB-MRC was Alan Weeds who taught me about the biochemistry of myosin and its sub-fragments, which I used in my studies. I was fortunate in those 2 years to combine biochemistry with helical reconstructions from electron micrographs that led us to hypothesize the steric blocking mechanism for tropomyosin-troponin function (Spudich et al., 1972), a hypothesis extended by low angle X-ray diffraction studies of muscle by Hugh (Huxley, 1972) and by Parry and Squire (Parry and Squire, 1973). The steric blocking model gained more and more experimental support as higher-resolution structures were solved (Lehman, 2016; von der Ecken et al., 2015; Tobacman, 2021). My training in structural biology at the LMB-MRC was pivotal for what I accomplished over the next 5 decades. Those 2 years in Cambridge at the LMB with Hugh provided me the structural biology tools to add to the interdisciplinary approach that was to define my entire career.

## With training in chemistry, physics, biochemistry, genetics, and structural biology, what’s next?

From Cambridge, I accepted a position as Assistant Professor of the new Department of Biochemistry and Biophysics at UCSF, chaired by Bill Rutter. It was 1971 and I had multiple ideas about important biological problems to apply my interdisciplinary training to, including the interesting developmental biology project that I had worked on earlier–what’s involved in the conversion of a bacterial vegetative cell into a dormant spore, and then back again to a vegetative cell when the correct environment is present. Whatever I decided to work on, the objective was to design experiments that would provide definitive answers to the pivotal questions at hand, regardless how difficult. Throughout my career, I have told my students and postdoctoral fellows to go for the decisive experiment, do not be afraid to fail, dream about your work, and be grateful for the privilege of carrying out creative research.

I wrote an NIH grant for the sporulation project while still in Cambridge, which was funded. By the time I arrived for my position at UCSF, however, my intuition led me to study two other fundamental unanswered questions in cell biology at the time: how the chemical energy of ATP hydrolysis brings about mechanical movement of muscle contraction, and what roles does a myosin-like motor have in nonmuscle cells. I called my NIH grants officer and explained that I had decided to work on something totally different. He asked me to send a one-page description of what I was doing to put in his file, and I used the funds awarded to study bacterial sporulation to study myosin instead–gone are those days!

Given the dictum *“You will never understand how a system works until you can reconstitute the functions of interest from purified proteins,”* where the primary myosin-driven functions of interest for muscle and nonmuscle cells are movement and force production, my key first goal was to develop a quantitative *in vitro* motility assay for movement of muscle actin driven by the molecular motor myosin. From my earlier experience I knew one could add myosin to a clean slide and form a monolayer of the motor protein on which actin filaments might move, but how does one visualize the actin filaments, which as individual filaments are too thin to be seen by conventional light microscopy. The first possible solution derived from my studies of actin from the cellular slime mold *Dictyostelium discoideum*, as described below.

My second goal was to develop a model organism to unravel the molecular basis of the myriad nonmuscle-cell movements that are clearly visible by light microscopy. In the late 1960s, Sadashi Hatano and Tazawa in Japan (Hatano and Tazawa, 1968), and Mark Adelman and Ed Taylor in the United States (Adelman and Taylor, 1969a; Adelman and Taylor, 1969b), had shown that actin and myosin are present in the acellular slime mold *Physarum polycephalum*. And as they recall in this issue (Pollard and Korn, 2023), Tom Pollard and Ed Korn found an entirely new type of myosin that changed the field in a dramatic way (Pollard and Korn, 1973a; Pollard and Korn, 1973b). Pollard and Korn were the initiators of what became an explosion of interest in unconventional myosins. Also in 1969 (Ishikawa et al., 1969), microfilaments associated with cell membranes were shown to be actin, by decorating them with the heavy meromyosin (HMM) fragment of myosin to give the characteristic arrowhead appearance that Hugh Huxley had shown for the muscle proteins in 1963 (Huxley, 1963). Non-muscle cell motility was on the verge of an explosion of activity.

At UCSF my laboratory explored *Neurospora crassa, Saccharomyces cerevisiae, Physarum polycephalum, Dictyostelium discoideum, Nitella axillaris, Strongylocentrotus purpuratus* and chick embryo fibroblasts (CEFs), none of which I had worked on in my previous training. The giant cells of the alga *Nitella* were particularly intriguing because of their striking intracellular cytoplasmic streaming that was visible under a simple light microscope, which was one of the first items I purchased for my own laboratory. Although not suitable for biochemistry or genetics, *Nitella* would assume an important role in my lab a decade later.

Our attempts to explore CEFs as a biochemical system led to an interesting finding. These cells were being worked on by Warren Levinson at UCSF. When we lysed the CEF cells growing on plates with the detergent Triton X-100, and viewed the plate by light microscopy, we noticed that a ‘ghost’ of each cell was still present, with its nucleus still apparent, somehow being held in its original position. Further electron microscopy revealed an extensive actin bundled network forming what we called a ‘cytoskeleton’ (Brown et al., 1976), a term I thought we coined, but Tom Pollard, a master of cell biology literature and a reviewer of this manuscript, pointed out that the term ‘cytoskeleton’ was developed in the 19th and early 20th centuries (Pollard, 1976; Frixione, 2000).

The slime mold *Dictyostelium* proved to be best for biochemical studies. Margaret Clarke, one of my first postdoctoral fellows, identified a myosin in *Dictyostelium* with properties similar to conventional muscle myosin (Clarke and Spudich, 1974), unlike the lower molecular weight *Acanthamoeba* myosin, named myosin-I, described by Tom Pollard and Ed Korn the year before (Pollard and Korn, 1973a; Pollard and Korn, 1973b). The conventional muscle myosin became known as myosin-II, and the *Dictyostelium discoideum* (D. d.) myosin that Margaret discovered was a myosin-II type. Later, Margaret (Meg) Titus (Titus et al., 1989; Titus et al., 1993) and John Hammer (Wessels et al., 1991; Urrutia et al., 1993; Hammer and Jung, 1996) identified multiple other forms of myosin in *Dictyostelium*.

## Reconstitution of functions of interest from purified proteins often requires expression of the proteins of interest in an appropriate cell system

In 1977, I joined the new Department of Structural Biology at Stanford, where we continued to expand on our work on D. d. myosin II. Modern biochemistry commonly includes using molecular genetics to express and purify proteins of interest in an appropriate cell system. Unfortunately, no myosin type has ever been able to be expressed in a functional form using bacteria as an expression system. We hoped we might be able to use *Dictyostelium* for this purpose, though *Dictyostelium* had not been used as an expression system before. In attempts to express a truncated form of D. d. myosin II in *Dictyostelium* cells, Arturo de Lozanne discovered unexpectedly that one can very efficiently target genes in *Dictyostelium* by homologous recombination (De Lozanne and Spudich, 1987). He disrupted the single copy D. d. myosin II gene and provided the first genetic proof that myosin II is required for cytokinesis. Dietmar Manstein, Meg Titus and Arturo then used a linear plasmid to knock out the D. d. myosin II gene (Manstein et al., 1989). *Dictyostelium* cells in suspension undergo normal cell division associated with the mitotic cycle. The D. d. myosin II knockout cells fail to divide in suspension and become large and multinucleated. On a surface, however, the knockout cells divide by a process we named *traction-mediated cytofission*, which is not associated with the mitotic cycle and might be how eukaryotic cells divided in early evolution. Importantly, the knockout cells were able to form pseudopodia and move along a surface, disproving the prevailing hypothesis that myosin II was needed for this process. Looking for an alternative force for driving cells forward, it soon became apparent that actin polymerization was the driving force (Theriot, 2000; Pollard and Cooper, 2009). While the D. d. Myosin II is not needed for general cell movement, it is required for directed cell movement, as occurs during *Dictyostelium* chemotaxis. *Dictyostelium* cells accumulate myosin II at their rear, inhibiting pseudopodia formation in that region, providing a force to detach the rear of the cell from the surface that the cell is moving on as the cell moves forward, and giving the cell the polarity it needs for directed cell motility (Liang et al., 2002).

Sometimes in research you are dealt a spectacular hand that you were not expecting. Suddenly, we had a eukaryotic cell that was entirely devoid of myosin II, and we could keep these cells alive by growing them on a surface where they underwent traction-mediated cytofission. We soon showed that we could rescue cytokinesis in suspension by replacing the myosin gene by introducing a copy of the D. d. myosin gene on a plasmid (Egelhoff et al., 1990). This was followed by rescuing cytokinesis with a GFP-tagged D. d. myosin II, which allowed us to quantify the movements of the myosin during various stages of the cell cycle and watch its accumulation in the pre-furrow region during mitosis (Moores et al., 1996). We also used the power of combining this cellular system with biochemical studies of purified proteins to understand the regulation of myosin thick filament formation by phosphorylation of the C-terminal portion of the myosin tail (Egelhoff et al., 1993; Sabry et al., 1997; Liang et al., 1999; Liang et al., 2002).

Another example of the power of combining biochemistry and cell biology is seen in the work of my graduate student William Shih. William took on the daunting task of creating a form of D. d. Myosin II to be used to carry out time resolved fluorescence energy transfer measurements on the myosin in various nucleotide states. This required removing existing cysteine residues from the myosin head domain and placing cysteine residues, which could be tagged with donor and acceptor fluorescent probes, at appropriate positions that would reflect the orientation of the so-called lever arm of the myosin head. But how would he know that the modifications he made left the myosin in a functional form? The answer is that he could express his altered myosin in the myosin null cell and make sure it could rescue cytokinesis. His studies were the first to demonstrate dynamically that the lever arm exists in different orientations depending on the nucleotide state in the active site (Shih et al., 2000).

Another stellar graduate student, Kathy Ruppel, used random mutagenesis to create 21 point mutations in the motor domain of D. d. myosin II to dissect structure/function relationships. She classified them into three distinct groups based on the ability to complement myosin null cell phenotypes: wild type, intermediate, and null. Biochemical analysis of the mutated myosins also revealed three classes of mutants that correlated well with the phenotypic classification (Ruppel and Spudich, 1996). Such an extensive mutational analysis of myosin function could only begin to be done by others later when myosins began to be expressed in Sf9 and mammalian expression systems. Kathy’s work coincided with the publication of the myosin head structure by Ivan Rayment and his colleagues (Rayment et al., 1993a; Rayment et al., 1993b; Schroder et al., 1993), which allowed her to also place the mutations in the context of the 3D structure of the myosin. This was a tour de force made possible because of the properties *Dictyostelium* and, of course, the exceptional talents of Kathy Ruppel.

Young investigators looking for a model eukaryotic cell to explore their chosen biological process of interest should consider adopting *Dictyostelium* (Egelhoff et al., 1991; Egelhoff and Spudich, 1991). I know of no other cell that has many of the properties of mammalian cells, can be easily grown in large quantities for expressing proteins for biochemistry, and offers the opportunity to meld biochemical results with cellular behaviors utilizing homologous recombination. *Dictyostelium* could be the organism of choice for modern biochemists and biologists, comparable to *Escherichia coli*, which served the biochemistry community so well in earlier decades.

**FIGURE 2:**
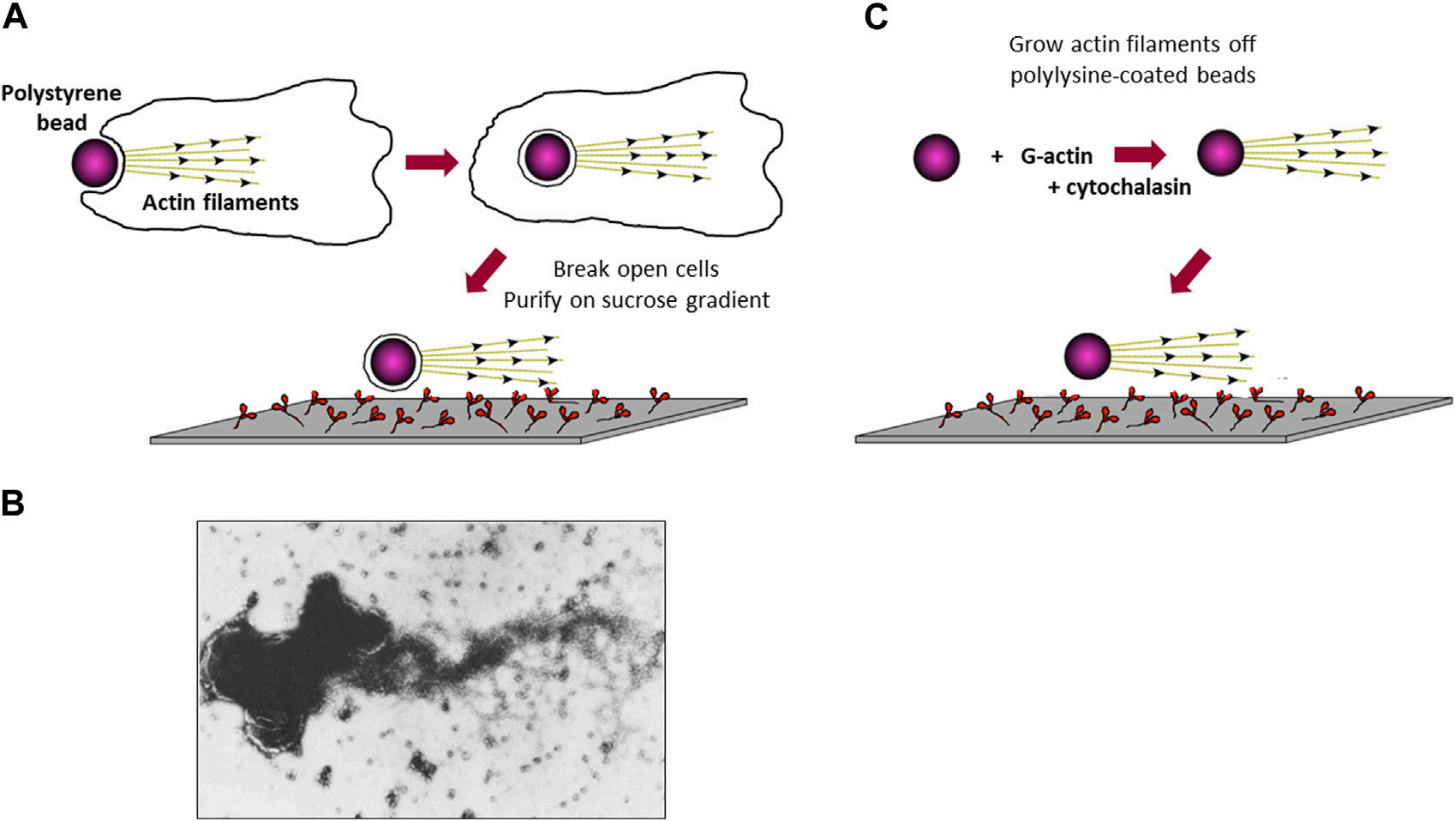
Attempts to visualize *in vitro* movement of actin-coated polystyrene beads along a myosin-coated glass slide. **(A)** Schematic drawing of a polystyrene bead being phagocytized by a *Dictyostelium* cell, followed by cell lysis and purification of the actin-coated polystyrene beads to test for movement on a myosin-coated slide. **(B)** Electron microscope image of a cluster of beads with a tuft of actin filaments projecting off its surface. The major protein in these preparations was shown biochemically to be actin (Spudich and Clarke, 1974). **(C)** Schematic drawing of actin grown off the surface of a polylysine-coated polystyrene bead, followed by testing for movement on a myosin-coated slide.

In the early days of my UCSF laboratory, I worked on identifying actin in *Dictyostelium* and showed that it is associated with the cell membrane. *Dictyostelium* is a highly phagocytic organism and readily engulfs polystyrene beads, turning the cell membrane inside out (Figure 2A). Actin filaments are polar, and this polarity can be visualized by decorating the actin filaments with myosin, which gives rise to an arrowheaded appearance every 36 nm along the filament. Thus, the actin filament has a barbed end and a pointed end. In muscle, myosin molecules move along each actin filament toward its barbed end (Huxley, 1963), which is anchored to the muscle Z-lines. Assuming actin filaments were similarly anchored to cell membranes at their barbed-ends, the phagocytized beads should have tufts of actin filaments emanating from them, which should move along a myosin-coated glass surface (Figure 2A). Thus, I enriched the phagocytized beads from cell lysates on a sucrose gradient and showed that they did indeed have actin filaments attached (Spudich and Clarke, 1974) (Figure 2B). When added to the myosin-coated slide, I observed some saltatory particle movements, meaning some phagocytic vesicles moved directionally over short distances! But these were rare and not quantifiable in a convincing way. The results, however, encouraged me to believe that it would be possible to observe actin moving along a myosin-coated surface when we understood more about the system, and when we found a good way to visualize the actin filaments.

In 1977 I was recruited to be the first faculty member to join the newly formed Department of Structural Biology at Stanford, headed by Lubert Stryer. In the next years we extensively characterized the actin-myosin system in *Dictyostelium*. Interestingly, inconsistent with Hugh Huxley’s swinging cross bridge proposal (Huxley, 1969), Toshio Yanagida (Yanagida, 1981), using polarized fluorescence microscopy to measure the angles of fluorescent nucleotides bound to myosin heads during muscle contraction, and the laboratories of Roger Cooke and Dave Thomas, using labeling of a sulfhydryl group in the myosin head domain with electron paramagnetic resonance (EPR) probes (Cooke et al., 1982; Cooke et al., 1984; Cooke, 1986), showed that the entire head domain does not change orientation during the power stroke of the contractile interaction between myosin and actin. It was more imperative than ever to develop a quantitative *in vitro* motility system to test the various models under consideration. In new experiments, we created actin filament coated beads, reminiscent of the *Dictyostelium* phagocytized beads I explored earlier, by nucleating actin filament growth off polylysine-coated polystyrene beads (Brown and Spudich, 1979). In the presence of cytochalasin D, which binds to the barbed-end of filaments and blocks actin monomer addition, actin filaments grow with their barbed-ends at the bead surface and pointed-ends projecting outward (Figure 2C). Unfortunately, these still did not show robust and convincing directed motion on myosin-coated surfaces.

**FIGURE 3:**
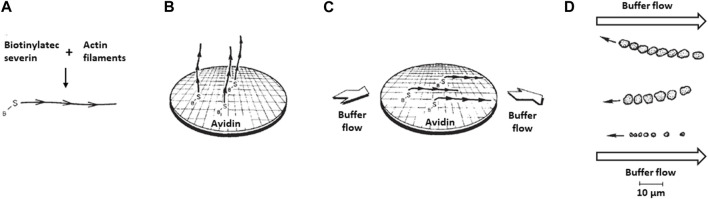
Movement of myosin-coated polystyrene beads on oriented actin filaments on a surface. **(A)** Biotinylated-severin is attached to the barbed-end of actin filaments. **(B)** The biotinylated severin bound actin filaments are attached to an avidin-bound surface. Depicted is a schematic drawing of an electron microscope grid placed on the glass slide before avidin coating to allow eventual examination by electron microscopy of the quality of the actin filament orientation after buffer flow across the slide. **(C)** Buffer flow across the slide orients the actin filaments. **(D)** Actual upstream movement observed of three different-sized clumps of myosin-coated beads along oriented actin filaments. The two larger bead aggregates were coated with D. d. myosin II and their positions are shown every 10 s, corresponding to a rate of movement of ∼0.5 μm s^-1^. The smaller bead aggregate was coated with skeletal muscle myosin and its position is shown every 2 s, corresponding to a rate of movement of ∼2 μm s^-1^. Figure adapted from (Spudich et al., 1985).

So, we reversed the components and attempted to watch myosin-coated beads move along oriented actin filaments attached to a glass slide. In 1981, Susan Brown and Keichi Yamamoto, postdoctoral fellows in my laboratory, had identified and purified *Dictyostelium* severin (Brown et al., 1982; Yamamoto et al., 1982), a protein that severs actin filaments, but more importantly, tightly binds the barbed-ends of actin filaments and can be tagged with a small molecule, as shown by Rona Giffard and Alan Weeds in my lab (Giffard et al., 1984). The idea was to use biotinylated severin to attach actin filaments by their barbed ends to an avidin-coated slide (Figures 3A, B). *Remember, the first protein put down onto a clean glass surface will form a monolayer, in this case avidin, and so the actin filaments will not bind nonspecifically and only bind through the avidin-biotin link*. Hence, biotinylated-severin bound to the barbed-end of actin filaments attach the actin filaments to the surface *via* their barbed-ends (Figure 3B), with the filaments being free to be oriented by buffer flow over the slide (Figure 3C). The actin filaments should then orient with the pointed-ends of the filaments downstream, and the myosin-coated beads should move upstream against the flow of the buffer. When we placed the myosin-coated beads on these actin-coated slides and added ATP we were disappointed to not see convincing robust directional movements. In retrospect, we did not have sufficient alignment of the actin filaments–we were not monitoring filament alignment at that time by electron microscopy, as we did later.

Then in 1982, Mike Sheetz joined my laboratory as a sabbatical visitor and was interested in improving the myosin-coated bead movement along severin-bound actin filaments oriented on a surface. It still was not working. This is when *Nitella* re-entered my lab. Since the early 1970s when we first explored *Nitella*, Yolanda Kersey, who was a student in Tom Pollard’s laboratory interested in myosin in plants, came to Norm Wessells’ laboratory in the Department of Biological Sciences at Stanford as a postdoctoral fellow to study the basis for motility in *Nitella*. Kersey and Wessels showed that actin cables in *Nitella* are well organized and continuous along the cytoplasmic face of organized chloroplast rows (Kersey and Wessells, 1976). Yolanda contacted me to get advice about using myosin to decorate the actin filaments to visualize their orientation. I happily supplied her with the myosin fragment HMM and advised her on the best conditions to use it to decorate the actin. Using HMM binding, Yolanda showed that the actin cables (filaments) are oriented in the direction consistent with the observed direction of movement of presumed myosin-coated vesicular elements (cytoplasmic streaming), the direction expected if a conventional myosin was involved (Kersey et al., 1976). Yolanda’s experiments were pivotal for what happened next in my laboratory.

At one late-night session at the laboratory with Mike, I suggested that we get some *Nitella*, the cylindrical cells of which can be a centimeter long and sufficiently wide to cut open longitudinally with iridectomy scissors. The cut open cell could then be pinned down by its four corners so that the oriented actin cables would be exposed. Furthermore, the right person to show us how to do this was a fellow faculty member in the Department of Structural Biology, Peter Sargent. Peter is a neurobiologist who was performing such surgical operations on similar-sized nerve axons. Peter showed Mike how to cut open the *Nitella*, Mike added the myosin-coated beads, and they moved steadily and unidirectionally along chloroplast rows in the very first experiment (Sheetz and Spudich, 1983)! The myosin coating the beads prevented the beads from binding other proteins, such as the actin cables themselves, and so they were free to move along the oriented actin filaments without a load. Peter Sargent’s help with *Nitella* was a key step in this pivotal experiment.

Mike then left my laboratory for Woods Hole with Eric Shooter’s graduate student Ron Vale to see if they could see myosin-coated beads move along tracks in squid axons. Together with Bruce Schnapp and Tom Reese, Ron and Mike found that myosin was not involved in this movement, as they originally assumed, and discovered and purified a new microtubule-based molecular motor which Ron named kinesin (Vale et al., 1985a; Vale et al., 1985b). Their discoveries depended on the principle that the first protein that a glass slide or polystyrene bead encounters forms a monolayer of that protein on the surface, which then keeps the surface from non-specifically binding to other proteins. Their discovery of kinesin energized the field and opened years of exciting work from their laboratories and many others.

Meanwhile, at Stanford I was working to eliminate the vagaries of the complex *Nitella* substratum and establish a totally-defined *in vitro* motility assay for myosin moving on actin–one must reconstitute the functions of interest from purified proteins. The *Nitella* experiment showed that the myosin-coated beads were functional and the actin filament orientation in our severin-based assay was undoubtedly the problem. I returned to the severin-based assay to try to get better orientation of the actin filaments, now using the electron microscope to judge orientation, and the expected upstream movement could now be clearly seen (Figure 3D).

Then, in January 1984, Steve Kron joined my laboratory as a graduate student, and his first task was to optimize this assay. With Steve’s prior bioengineering expertise in fluid mechanics and viscometry, we got good movement of myosin-coated beads on oriented actin filaments (Spudich et al., 1985), the first myosin movement assay with purified proteins. Importantly, this assay established that one can achieve several μm s^-1^ velocity, approaching the velocity of contraction of unloaded muscle contraction, with nothing more than purified actin, myosin, and ATP. The assay, however, was too complicated to be used widely.

## A pivotal observation by Toshio Yanagida led Steve Kron to develop the assay that became standard in the field

In 1984, Toshio Yanagida and his students reported the seminal finding that one can visualize individual actin filaments labeled with rhodamine-phalloidin by fluorescence microscopy (Yanagida et al., 1984). This was fantastic because it allowed us to return to the myosin-coated surface concept that we had tried earlier, but now we could see the actin filaments directly by fluorescence microscopy! In Toshio’s 1984 paper (Yanagida et al., 1984), he and his students demonstrated effects on the Brownian motion of actin filaments in solution when myosin and ATP were added, an assay for monitoring actin-myosin interactions, but not an assay for making velocity measurements.

**FIGURE 4:**
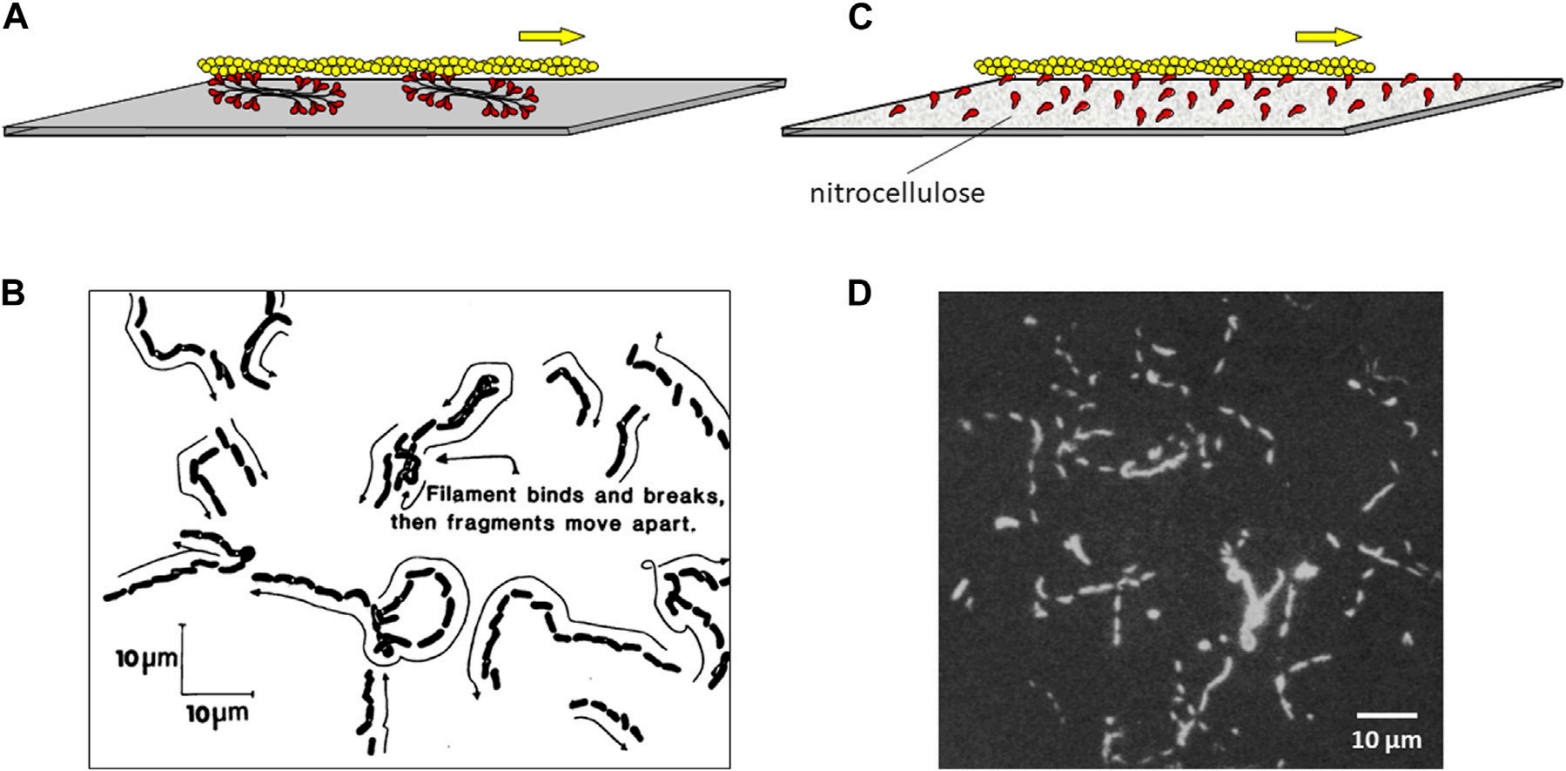
Movement of fluorescent actin filaments on a myosin-coated surface. **(A)** Schematic drawing of an actin filament (yellow) moving across a lawn of myosin bipolar thick filaments (red) coating a microscope slide. **(B)** Actual trajectories of actin filaments moving on myosin filaments as diagrammed in **(A)**. Skeletal muscle actin filaments moved on skeletal muscle myosin attached to the slide over 38 s. Their positions are indicated at successive short intervals as they appeared on the video monitor. The average rate of movement in multiple experiments was ∼3 μm s^-1^. Figure reproduced from (Kron and Spudich, 1986). **(C)** Schematic drawing of an actin filament (yellow) moving across a lawn of purified S1 myosin heads (red) coating a microscope slide, which was pretreated with nitrocellulose. **(D)** Stroboscopic photograph of movement of fluorescent actin filaments moving on papain derived skeletal muscle S1. A photographic shutter was used to illuminate the filaments for 1 s every 4 s for a total of 12 s. The scale bar is 10 µm. Thus the average rate of movement was ∼1–2 μm s^-1^. Figure reproduced from (Toyoshima et al., 1987).

Determined to find an assay that was easier than the myosin-coated bead assay, Steve Kron coated a glass slide with purified skeletal myosin thick filaments and then rinsed the slide with BSA to make sure any remaining nascent surface area was saturated with protein. He then added fluorescently labeled actin filaments and ATP. The experiment worked the first time (Kron and Spudich, 1986) (Figures 4A, B), and the rest is history. Since the surface was covered with myosin plus BSA, the actin was free to move along the myosin-coated surface without showing non-specific binding to the surface. The Kron assay is so simple and so robust, that it has naturally assumed a prominent position in actin-myosin biology and muscle contraction work throughout the world. Steve’s experiment has been said to be transformative in the field of muscle and actin-myosin-based nonmuscle motility (Rall, 2014), the type of accolade every PhD student and scientific advisor welcomes.

The power of having developed a quantitative assay with purified proteins for the function of interest, movement in this case, was made immediately obvious by my postdoctoral fellow Yoko Toyoshima, who together with Steve Kron, Elizabeth McNally, and others in my laboratory showed that the globular head, or subfragment 1 (S1), of myosin is the motor domain (Toyoshima et al., 1987). In these experiments, Yoko tried saturating the slide surface with a variety of reagents before adding the S1 because adding the S1 directly to the clean slide did not work well, presumably because the S1 bound to the clean glass in an orientation that was not favorable for actin interaction. This was not a problem for the myosin thick filaments bound to the slide, because the structure of the thick filament assures there is always an array of myosin heads oriented away from the slide and able to interact with actin (Figure 4A). Nitrocellulose solved the S1 problem (Figures 4C,D). Something about adding the S1 to a nitrocellulose-coated slide allowed a sufficient number of S1 molecules to be oriented properly to drive actin filament movement smoothly across the surface for prolonged periods. This result eliminated competing theories to the swinging cross-bridge hypothesis and focused research on the S1 head to understand how the myosin family of molecular motors works. Almost 2 decades of work finally led us to the early goals I had set for my lab in the early 1970s.

## But what about force, the other function of interest that needed to be reconstituted from purified proteins?

With the Kron assay solving the need for a robust and simple *in vitro* assay for measuring the velocity of movement of myosin along actin, the next step was to find a way to measure the force that myosin molecules produce when they interact with actin in a totally reconstituted purified protein system. This was first accomplished in 1988 by Toshio Yanagida and his students, who developed a technique for manipulating a single actin filament using a fine glass needle to measure and exert force on it by a small number of myosin molecules (Kishino and Yanagida, 1988). They extended this technique and measured displacements and forces with nanometer and piconewton resolutions in the millisecond time range (Ishijima et al., 1991). Details of this glass needle approach was described in 1996 (Ishijima et al., 1996). However, it was imperative to clearly visualize what a single myosin molecule does when it interacts with actin because of disagreements at the time about how far myosin moves along actin upon hydrolyzing one ATP molecule (the step size). Experiments from Toshio’s laboratory reported the step size to be > 40 nm (Yanagida et al., 1993; Saito et al., 1994). This number would rule out the conventional swinging cross bridge model of muscle contraction, whereas our experiments had suggested a smaller step size of about 10 nm, which was compatible with the swinging cross bridge model (Toyoshima et al., 1990; Uyeda et al., 1990; Uyeda et al., 1991). Toshio and I debated about these results at scientific meetings and in published papers for more than a decade during the years 1980–1995, while maintaining a close friendship and respect for one another. In the early 1990s we agreed, over a glass of wine, that what was needed was to watch what a single myosin molecule does when it interacts with a single actin filament. Steve Kron played an important role in the next step for my laboratory.

My colleague Gil Chu, in the Department of Biochemistry, imagined that Steve Kron in my lab might be interested in helping his brother, Steve Chu, who hoped to exploit optical traps to manipulate biological molecules. Steve Chu had recently arrived at Stanford from Bell Labs where he completed his work on cooling and trapping atoms for which he later received the Nobel Prize. He was excited to take on new challenges. Steve Kron and Steve Chu found time to work together in the evenings and learned to image single DNA molecules held in flow by optically trapping beads tethered to the ends. This was Steve Chu’s entree to molecular biology and his initial connection to my laboratory.

**FIGURE 5:**
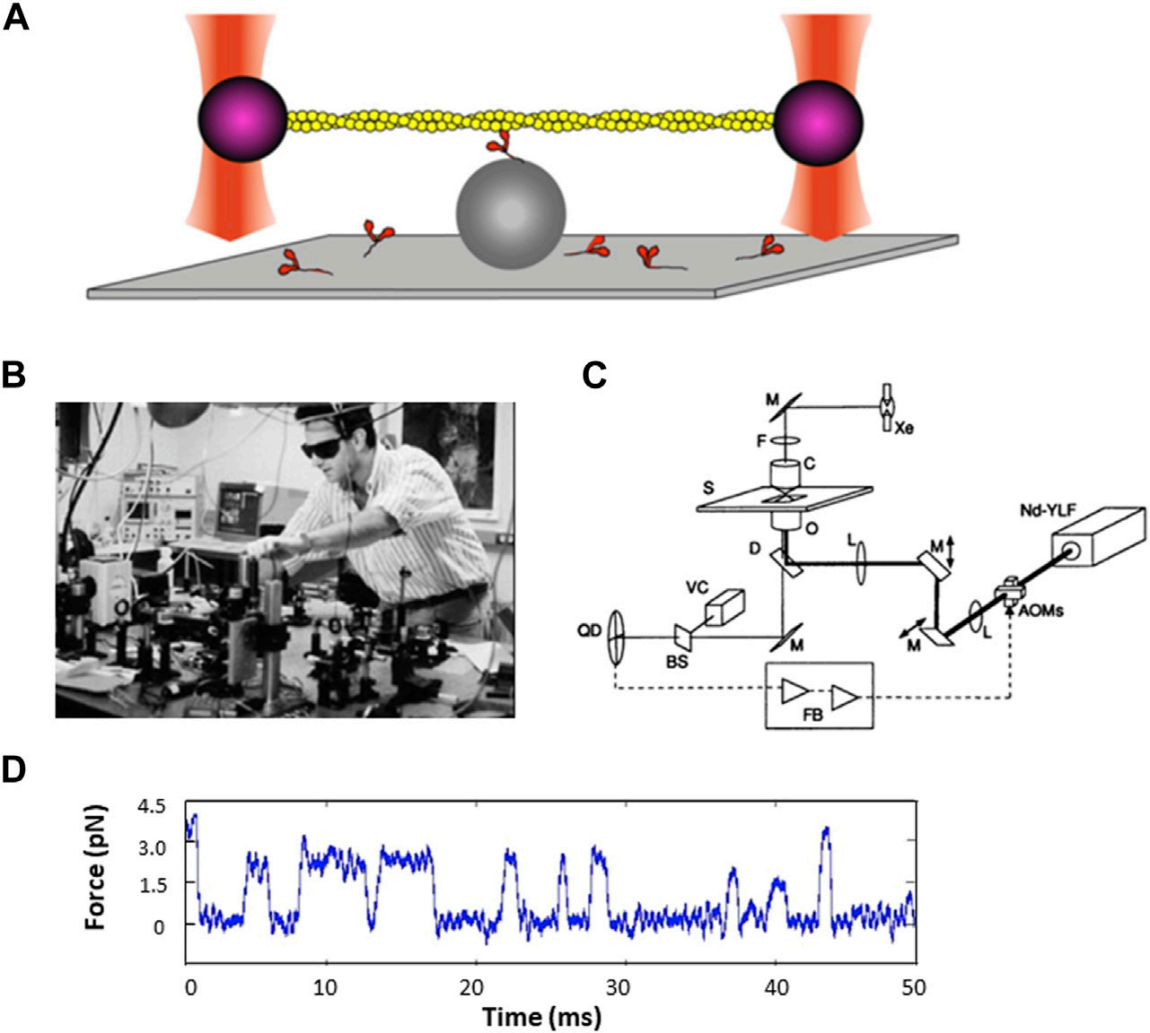
Force measurements using the dual beam laser trap. **(A)** Schematic drawing of a single fluorescent actin filament (yellow) with a 1-µm polystyrene bead (purple) attached at each end of the actin filament, with each bead being held by an independently controlled laser trap (red). The filament is lowered onto another polystyrene bead (grey) fixed to a microscope slide, with a single myosin molecule on top. **(B)** Photograph of Jeff Finer building the dual beam laser trap in the Beckman building basement. Reproduced by permission. **(C)** Schematic diagram of the feedback-enhanced laser trap system. The thick solid line represents the laser beam path; the thin solid line represents the imaging path. Brightfield illumination from a xenon arc lamp (Xe) is used for visualizing the sample with a video camera (VC) and used to project an image of a trapped bead in the specimen plane (S) onto a quadrant photodetector (QD). Two orthogonal acousto-optic modulators (AOMs) are used for rapidly deflecting the laser beam before it enters the back aperture of the microscope objective (0). Additional optics include lenses (L), mirrors (M), dichroic filter (D), interference filter (F), beamsplitter (BS), and microscope condenser (C). The second trapping beam and fluorescence imaging pathways are not shown for simplicity. The quadrant detector output (dashed line) can be processed by feedback electronics (FB) and used to drive the acousto-optic modulators. Figure reproduced from (Finer et al., 1995).^1^ **(D)** Sample trace of force transients as a function of time.

A new graduate student in my lab, Jeff Finer, and a sabbatical visitor, Bob Simmons, a well-known British investigator with extensive experience in developing specialized equipment with feedback systems for measuring force transients in muscle fibers, wanted to adapt the Kron *in vitro* motility assay to the single molecule level. Jeff, Bob, and I wanted to develop a laser trap for trapping a single bead attached to the barbed-end of an actin filament and have the filament be acted upon by a single myosin molecule on a surface, a modification of the Kron motility assay. Jeff and Bob tried several different geometries with different ways to raise myosin molecules above the surface, such as engineered surfaces with ridges, but nothing was working. Then one night Jeff had a dream in which a dumbbell consisting of a single actin filament with a polystyrene bead attached at each end, each bead being trapped by its own laser beam, was lowered onto a single myosin molecule raised off the glass slide by another bead fixed to the surface (Figure 5A).

With Steve Chu’s help, Jeff and Bob built the first dual beam laser trap in Steve’s space in the Department of Physics in the Varian building, using a Zeiss microscope as its base. They started with a single-beam gradient optical trap with a high-resolution photodiode position detector to show that an optical trap can be used to make quantitative measurements of nanometer displacements and piconewton forces with millisecond resolution (Simmons et al., 1993; Simmons et al., 1996). For the dual beam trap, they started with a quadrant detector (QD) to look for possible myosin-driven movement of actin attached to a bead at each end, and then, at Steve Chu’s suggestion, they added acousto-optic modulators (AOMs) to bring feedback into the system. They also added motors to move one of the beams to eliminate having to move knobs for the mirrors and the stage. But that trap was on an upper floor of the Varian building and there was too much vibrational noise for the measurements we wanted to make with our actin-myosin system, so Jeff built another dual-beam trap in the basement of the Beckman building, on solid ground and just downstairs from my laboratory. Bob had returned to England but continued to help from afar. Jeff made several pivotal changes to the new dual-beam trap in Beckman, including getting rid of a microscope base and switching to a custom-made open optics system where he had better control over all the parts, and adding more motors to control the traps and the stage, which dramatically increased the efficiency of catching actin filaments (Figures 5B, C). He also improved nearly every other component including a laser that did not need a lot of water cooling. Even so, there was still too much noise to pick up the step size or force measurements produced by a single myosin molecule.

An obvious issue was the airflow in the room, and one day I suggested to Jeff that he buy enough posterboard to cut and tape together to cover all the optics to isolate the path of the laser beam from the general room airflow. This worked! The twenty dollars’ worth of posterboard added to the $100,000 instrument Jeff had built allowed us to see individual force and step size transients generated by a single myosin molecule interacting with a single actin filament. That trap gave us the first convincing step size and force traces that allowed us to conclude that a single myosin molecule produces force in the single digit pN range and displaces an actin filament by about 10 nm for each interaction (Finer et al., 1994) (Figure 5D). Justin Molloy was an early important contributor in this area and described an analysis method that suggested a somewhat shorter step size of about 4 nm (Molloy et al., 1995). Together with Claudia Veigel, as well as independently, Justin and Claudia have made numerous important contributions toward developing single molecule laser trap methodology and understanding how myosin motors work (for reviews, see (Knight et al., 2001; Ruegg et al., 2002; Ruegg et al., 2002; Molloy and Veigel, 2003; Veigel and Schmidt, 2011; Batters and Veigel, 2016)).

This was the time that Amit Mehta, a graduate student in the Physics Department, joined my laboratory. Over the years, I have hosted several Physics graduate students in my laboratory. They carried out their PhD work in my lab and got their degrees from the Department of Physics. Amit, like all the others, was an outstanding student, brought his strong physics background to our effort, and helped us understand the strengths and limitations of our trap measurements (Mehta et al., 1998a; Mehta et al., 1998b). He also contributed new insights such as the ability to detect single-molecule interactions using correlated thermal diffusion (Mehta et al., 1997).

Meanwhile, Steve Chu’s students were carrying out single molecule analyses of fluorescently labeled DNA (Perkins et al., 1994a; Perkins et al. 1994b; Perkins et al., 1995; Smith et al., 1995; Perkins et al., 1997). Just as Jeff Finer and Bob Simmons from my lab spent months working in Steve Chu’s lab to build the first dual beam trap, four of Steve’s physics students spent more than a year working in space in my lab in Beckman generally soaking up biochemistry and molecular biology principles and specifically learning how to prepare DNA, ligate λ-phage DNA molecules together to make sufficiently long DNA molecules for their biophysical studies, and biotinylate one end of the DNA molecule and attach it to a streptavidin-coated polystyrene sphere to be manipulated in an optical trap. These students included Steve Quake, Tom Perkins, and Doug Smith, all of whom went on to establish careers interfacing physics and biology. What made my collaboration with Steve Chu so powerful was that our students spent considerable time in each other’s environments, and this led to the interdisciplinary program in biosciences, bioengineering, and biomedicine at Stanford called Bio-X (https://biox.stanford.edu/; for early history, see Supplementary Information).

Another key founder of single molecule measurements was my former postdoctoral fellow Steve Block (note there are many Steves in this story!). After contributing importantly to domains of myosin involved in force production in my lab (Hynes et al., 1987), in 1987 Steve left for his first independent position as a staff scientist at the Rowland Institute in Cambridge, MA and a Lecturer at Harvard. There he began developing a single beam laser trap for measuring the step size of the processive kinesin molecule (Block et al., 1990). Mike Sheetz and Scot Kuo at Duke University also developed a single beam laser trap and reported force measurements by a single kinesin molecule (Kuo and Sheetz, 1993). The trap that Steve Block and his colleagues described in their 1993 Svoboda et al. paper (Svoboda et al., 1993) was groundbreaking, with precise and accurate measurements of 8-nm steps of kinesin along a microtubule. Later, at Stanford, Steve and his colleagues took single molecule mechanics to an incredibly high level of resolution and precision and applied it not only to kinesin but to RNA polymerase as well (Shaevitz et al., 2003; Abbondanzieri et al., 2005; Greenleaf and Block, 2006; Block, 2007; Guydosh and Block, 2007; Herbert et al., 2008; Clancy et al., 2011; Andreasson et al., 2015).

In 2015, Jongmin Sung along with others in my lab developed the technique of harmonic force spectroscopy (HFS) using the dual beam trap (Sung et al., 2015; Sung et al., 2017). This method measures the force dependence of myosin interactions with actin by oscillating the stage during their attachment. HFS has the advantage that the force is applied to the myosin rapidly and no feedback loops are required. This approach has proved very useful. For example, Chao Liu in my lab led experiments using HFS to measure the detachment rate of single molecules of human β-cardiac myosin and its load dependence, and showed that both can be modulated by small-molecule compounds and cardiomyopathy-causing mutations (Liu et al., 2018).

In summary, Steve Block’s single beam rendition of laser traps and our dual beam trap approach for single molecule analyses have been adopted worldwide, and a new field of single molecule analysis has flourished and contributed to many new discoveries. We and others have used the dual-beam laser trap to study multiple myosin motors (for reviews, see, for example, (Mehta et al., 1999a; Rocket al. 2000; Warshaw, 2004; Dantzig et al., 2006; Sun and Goldman, 2011; Karagiannis et al., 2014; Greenberg et al., 2017). It was using the dual beam laser trap that allowed us, for example, to show that myosin V and myosin VI are processive motors (Mehta et al., 1999b; Rief et al., 2000; Rock et al., 2001) and to examine details of how they step along actin (Purcell et al., 2002; Liao et al., 2009; Dunn et al., 2010; Elting et al., 2011). Taro Uyeda, Alex Dunn, and Zev Bryant were key postdoctoral fellows who used the Kron *in vitro* motility assay, the dual beam laser trap, and other single molecule approaches to provide conclusive evidence for the swinging lever arm mechanism for myosin-based movement (Uyeda et al., 1996; Bryant et al., 2007; Dunn and Spudich, 2007).

Particularly powerful is combining the *in vitro* motility assay with laser-trap measurements to reveal mechanisms of myosin stepping. An example is Myosin VI, which was the biggest challenge to the lever-arm hypothesis. This unusual myosin takes very long (∼36-nm) steps (Yanagida and Iwane, 2000; Rock et al., 2001) despite having a very short light-chain-binding domain which in other myosins almost certainly acts as a lever arm to amplify mechanical movement. By mapping the step sizes using the laser trap and the velocities and direction of movement of four different constructs of myosin VI (Bryant et al., 2007) onto the known poststroke structure of the motor (Menetrey et al., 2005), it became clear that myosin VI does indeed conform to the swinging lever arm hypothesis. In this case, the lever arm strokes through an angle of ∼180° to give rise to a large mechanical stroke of ∼20 nm. The remaining ∼16 nm derives from diffusion of the free head in search of an appropriate actin-binding site. These results drove home the power of the *in vitro* motility assay and single-molecule analysis to reveal detailed structural information about functionally important mechanical transitions in proteins and solidified the swinging-lever-arm hypothesis as a general mechanism used by the myosin family.

The *in vitro* motility and dual beam laser trap assays also proved fundamental for understanding the molecular basis of hypercontractility in individuals having hypertrophic cardiomyopathy (HCM), a genetic disease often caused by mutations in human β-cardiac myosin, the molecular motor that drives systolic heart contraction. A key collaborator in my laboratory in these studies is Kathy Ruppel, and Suman Nag, Darshan Trivedi, and Masataka Kawana have played major roles (Nag et al., 2017; Trivedi et al., 2018; Trivedi et al. 2020; Trivedi et al. 2020; Kawana et al., 2022). Other key collaborators in our HCM studies are Leslie Leinwand, University of Colorado, Anne Houdusse, the Curie Institute, and Dan Bernstein and Euan Ashley, Stanford University School of Medicine. HCM hypercontractility primarily derives from a mutation-induced increase in the number of myosin heads accessible for interaction with actin (Spudich, 2015). This understanding led to the development of a first-in-class drug, mavacamten, which is an inhibitor of human β-cardiac myosin (Anderson et al., 2018; Rohde et al., 2018). Mavacamten normalizes the power output of HCM patients by reducing the number of myosin heads accessible for interaction with actin, simply reversing the effect of the HCM mutations.

## Conclusion

The *in vitro* motility and dual beam laser trap assays, as well as other related techniques (Churchman et al., 2005; Churchman et al., 2006; Dunn and Spudich, 2007; Mortensen et al., 2015), which were developed by talented students, postdoctoral fellows, and sabbatical visitors in my laboratory over the years, were based on the ‘mantra’ drilled into me in my graduate student days, *“one must reconstitute the functions of interest from purified proteins.*” These assays have been fundamental to the work on myosin molecular motors from my laboratory and many others in the field. As emphasized in this article, all discoveries depend on a cast of individuals over a long period of time. Such is the nature of scientific discoveries, as well as of other areas of creative endeavors. I thank the editors for giving me the chance to acknowledge those who played important roles in prominent discoveries that came from my laboratory in the latter part of the 20th and early part of the 21st centuries.

## Footnotes

1 This article was published in Biophys. J., Vol. 68 (4 Suppl. l), Finer, J. T., Mehta, A. D., and Spudich, J. A., Characterization of single actinmyosin interactions, 291S–297S, Copyright Elsevier (1995).
