# Supplementary material for: One must reconstitute the functions of interest from purified proteins
Source: Front Physiol. 2024 May 17;15:1390186. doi: 10.3389/fphys.2024.1390186 (PMC11140241; doi:10.3389/fphys.2024.1390186)
Supplement: Supplementary file 1 [file DataSheet1.PDF]

Supplementary Information

The early history of the development of the Bio-X Program at Stanford 1997-2001

James A. Spudich[1,#]

[1] Department of Biochemistry, Stanford University School of Medicine, Stanford, California 94305, United States
[#] Correspondence: jspudich@stanford.edu (J.A.S)

The editors have requested that I expand on my references to Bio-X in the main text, since the early history is not found on the Stanford Bio-X website. This grass roots effort was initiated by Steve Chu in the Department of Physics at Stanford, who had just received the Nobel Prize for his work on cooling and trapping atoms. Our collaboration had brought Steve prominently into biology, which resulted in his being recruited to another university to form a new research institute that would combine the physical and biological sciences. Rather than move, he decided that Stanford had all the elements to set up an interdisciplinary program and called me, Lucy Shapiro, founding Chair of the Department of Developmental Biology in the School of Medicine, and Richard Zare, Professor of Chemistry in the School of Humanities and Sciences, together on a Saturday to discuss the idea. All four of us were already using an interdisciplinary approach in our own research. The concept was to develop an interdisciplinary Program in Biosciences, Bioengineering, and Biomedicine involving the Schools of Medicine, Engineering, and Humanities and Sciences. The name was too long, and Richard Zare suggested it be called Bio-X, signifying bio-everything. There was a lot of collective enthusiasm at that Saturday meeting, and we decided to write a short ~5-page proposal and sent it to Stanford President Gerhard Casper and Provost Condoleezza (Condi) Rice to get their thoughts.

Condi responded quickly expressing her and Gerhard's high level of interest, and Steve and I met with her to explain that one thing that made our collaboration so powerful was that our students spent considerable time in each other's environments and that such an opportunity should be available broadly across the Schools of Engineering, Medicine, and Humanities and Sciences. Condi asked us to assemble a group of faculty representing the various constituents to work together to write a 'white paper,' describing our suggestions for specific plans for the initiative. The white paper included the suggestion that the Director of Bio-X should rotate among the faculty, as is common for the Chairs of many basic science departments on the campus, so that no one would jeopardize their own research programs by taking on this role for a prolonged period, and to allow representatives from the different Schools to be in this leadership position. We presented the white paper to Condi Rice and Gerhard Casper in 1998.

The plan was approved, and I was asked to be the first head of Bio-X, working closely with the Vice Provost and Dean of Research Charles Kruger, with whom I had nearly daily interactions that often included the weekends for the entire four years of my involvement. On day one, I suggested to Charles that we create an Executive Team, consisting of a representative from each of the three Schools involved, to work closely together to implement the initiatives, with Steve Chu of course, representing the School of Humanity and Sciences. Charles approved the idea and asked me to make recommendations for the representatives of the other two Schools. After some research, I recommended Channing Robertson from the School of Engineering and, recognizing the importance of having clinical representation on the Executive Team, I recommended Bill Mobley, Chairman of Neurology in the School of Medicine. This was approved and Steve, Channing, Bill and I worked closely together and with Charles Kruger for four years to facilitate the plans described in the white paper, with input from numerous faculty colleagues throughout the three Schools. Alice Gast, Associate Chair of the Department of Chemical Engineering at Stanford, became heavily involved, joined the Executive Team, and became an important contributor. My talented personal assistant in the Department of Biochemistry, Fiona Sincock, began devoting all her time to help me with the Bio-X initiative, and Beth Kane, recommended by Eugene Bauer, Dean of the School of Medicine, became the first Executive Director. Beth and Fiona worked closely with me tirelessly for the entire four years of my involvement and were heavily involved in the execution of all the initiatives that we established during that period.

The Executive Team reported our progress monthly to the Deans of the three Schools, Eugene Bauer, School of Medicine, Malcolm Beasley, School of Humanities and Sciences, and John Hennessy (until 1999) and then Jim Plummer when Hennessey became Provost, School of Engineering, and sought their counsel. I was fortunate to have the continuity throughout my four years of service of these highly supportive deans, none of whom tried to influence our grass roots initiatives with School politics. Our first task was to seek out and get to know key faculty from the many relevant Departments in all three Schools and to network to identify those who were interested in joining this interdisciplinary initiative. The interest was wide indeed, and hundreds of faculty signed on to be members of the Bio-X Program. Today the Bio-X membership is over 1000 faculty!

In the first year we established several important initiatives, including an interdisciplinary-focused seminar series, overseen for many years by Channing Robertson, and the establishment of multiple core facilities on campus for everyone's use, ranging from setting up microarrays for RNA analyses to X-ray crystallography. For everything we set out to do, faculty stepped up eager to help. Chaitan Khosla, Professor of Chemical Engineering and Chemistry, volunteered to organize the core facility initiative. We established an ongoing, now on round 12, Bio-X "Interdisciplinary Initiatives Seed Grants" opportunity, given out every other year. It started with a fund of $3M and has grown to $4M every other year. Applications are solicited from Stanford investigators working together across disciplines. For the seed grants initiative, I asked Harvey Cohen, Chairman of the Department of Pediatrics at Stanford University and Chief of Staff at Lucile Packard Children's Hospital, to head a grants review committee, and he and I identified and invited a dozen faculty from across the three Schools to serve. The first meeting was

fascinating because most of the faculty in the room did not know one another. The entire meeting involved everyone giving a description of who they were and what their research entailed. The excitement in the room was palpable, and it was clear that the impact of Bio-X was going to be profound.

The planning of a physical hub for Bio-X was high on our list. The Executive Committee worked closely with the architects to establish details of the design for the hub for Bio-X, the Clark Center, which is in the geographical center of the three Schools. We wanted to convince Norman Foster, known for his open architectural designs lending themselves to collaborative endeavors, to design the building. He did not immediately accept, and I suggested to Charles Kruger that we go to London and explain to him in person what our vision was. Charles agreed, and he, Channing Robertson, Alice Gast and I flew to London where we were hosted by Norman Foster and his team for three days. We came back with his agreement and a rough sketch made by him, which closely resembles the Clark Center as it is today. It was important that the Clark Center be seen as a hub, with more than 500 relevant faculty on campus signing on as members of Bio-X with the pledge to promote interdisciplinary sciences. Therefore, I made certain that a large restaurant be established on the ground floor of the Clark Center specified to have long rectangular tables, reminiscent of those in the 6th floor canteen of the MRC-LMB labs in Cambridge mentioned in the main text of this review, where investigators and their students and postdocs from everywhere on the campus would assemble and have important chance encounters with those from other disciplines. At one point, John Hennessey, who in October of 2000 became the 10th Stanford President, told me that they were short $10M and were going to have to cut the restaurant from the plan. I immediately responded that he should cut everything else and build the planned restaurant on the site, given its essential role in bringing people together from all the different scientific disciplines on the campus. He smiled with understanding and said, "OK, we will find the money." I and members of the Executive team worked weekly and some weeks daily with the architects to assure that all such details of the Clark Center met the scientific and collaborative goals of the Bio-X program. Key faculty from the three Schools who were potential occupants of the Clark Center were also involved. We saw the groundbreaking and establishment of the foundation and pillars of the building go up before our time of involvement ended.

Importantly, we were charged with identifying ~22 faculty from nearly as many departments from the three Schools to be the first occupants of the Clark Center. These individuals were recommended on the basis that they had a mindset for sharing space, equipment, and ideas, and communicating back to their respective Departments to assure that Bio-X was a campus-wide Program with the Clark Center serving as its hub. It was a bold and difficult exercise, but in 2001 our list was presented to John Hennessey, provost John Etchemendy, who succeeded Hennessy to the post in 2000 as Hennessey became Stanford's President, and the Deans of the three Schools. The initial inhabitants of the Clark Center, who set the tone of the Clark Center environment, were defined, along with the beginnings of specific interdisciplinary programs that brought these faculty together into subgroups with specific joint scientific goals. When asked what specific discoveries we hoped for, I have been quoted as saying, "If we knew that, we would have failed!" The idea was that new, exciting, and often not predicted directions would

emerge from the daily interactions that would occur between scientists, especially students and postdocs, from different disciplines. The remaining ~20 faculty or more were to come from new recruits who would identify with and be attracted by the opportunity to work in such an interdisciplinary environment. Among the first to be excited by this opportunity and be recruited to Stanford were Steve Block from Princeton, Steve Quake from Cal Tech, and my postdoctoral fellow Zev Bryant.

In 2002 Matt Scott became the second Director, and in his 5-year term, among other things, he oversaw the completion of the Clark Center, helped execute the move of the investigators from their current locations to the hub, and worked with faculty to establish specific programs of interdisciplinary research. In 2004, Heideh Fattaey was hired and eventually became Executive Director of Finance, Operations, & Programs, and continues to serve. She deservedly has been referred to as 'the soul of Bio-X' and received the 2011 Marshall D. O'Neill Award, which honors staff members who have made outstanding contributions to Stanford's research mission. I am sorry that I never had the pleasure of working directly with Heideh. Bio-X as we know it would not exist without her. In 2007, Matt left Stanford to become President of the Washington, D.C.-based Carnegie Institution for Science. Then Stanford made a hire that was transformational and breaking from our original recommendation of ~5-year terms for the Director. In 2007, Carla Shatz was recruited from Harvard to become the third Director, and under her 17-year leadership, Bio-X has blossomed and has lived up to the dreams we all had 25 years ago.

Acknowledgements

I thank Carla Shatz, Steve Chu, and Lucy Shapiro for useful comments and edits.
